# Impact of varicella vaccine on nosocomial outbreaks and management of post exposure prophylaxis following in a paediatric hospital

**DOI:** 10.1371/journal.pone.0251496

**Published:** 2021-05-20

**Authors:** Angela Gentile, Norberto Giglio, Maria Florencia Lucion, Ana Clara Martínez, Natalia Pejito, Maria del Valle Juarez

**Affiliations:** 1 Epidemiology, Ricardo Gutiérrez Children’s Hospital, Buenos Aires, Argentina; 2 Diego Azul Regional Maternity Hospital, Buenos Aires, Argentina; Erasmus MC, NETHERLANDS

## Abstract

**Introduction:**

In 2015, varicella vaccine was introduced to the National Immunization Programme in a one-dose regimen for infants aged 15 months. The aim of this study was to describe and compare the epidemiologic characteristics, management strategies and costs of varicella outbreaks in Ricardo Gutierrez Children’s Hospital (HNRG) from 2000 to 2019, before (PreV period) and after (PostV period) the introduction of the varicella vaccine.

**Methods:**

A retrospective, analytic study of the impact of nosocomial varicella outbreaks at the HNRG, based on active epidemiologic surveillance. We compared nosocomial varicella outbreaks rates (per 10,000 discharges) between PreV and PostV, excluding the intervention year (2015).

**Results:**

During PreV, an average of 15.87 (13.91–18.02) outbreaks per year was observed and in PostV 5.5 per year (3.44–8.32). Outbreaks adjusted by all cause discharges showed a reduction of 59.13% (-36.68%, -73.62%) after vaccine introduction.

Considering that in PreV the average of susceptible cases per outbreak was 5.0 and in PostV 7.8, with a cost per susceptible of AR$ $6,522 (80.27 USD) PreV and 6,708 PostV the economic impact on the reduction of outbreaks after the introduction of the vaccine, showed an estimated average savings per year of AR$ -252,128 AR$ (-3,103.11 USD).

**Conclusions:**

The number of annual varicella hospital outbreaks at the HNRG decreased significantly after varicella vaccine was introduced to NIP in Argentina with a relevant reduction in terms of costs.

## Introduction

Varicella is an acute, self-limited infection caused by varicella zoster virus (VZV) [1]. Although disease distribution is global; countries with temperate climates such as Argentina have seasonal fluctuations with incidence peaks occurring in late spring. Outbreaks often occur in hospitalized patients, healthcare workers, in schools and military staff. In 2015, the Argentinian Ministry of Health introduced the varicella vaccine to the National Immunization Programme (NIP) in a one-dose regimen for infants aged 15 months [2].

At the Ricardo Gutierrez Children’s Hospital (HNRG), a tertiary level medical center in Buenos Aires City, epidemiologic surveillance program of nosocomial varicella outbreaks has been conducted since 2000. Since then, over 200 outbreaks have been controlled implementing different strategies including ward closure, administration of antiviral agents, vaccination and use of varicella-zoster immune globulin (VariZIG) or intravenous immune globulin (IGIV). These measures helped prevent secondary cases and complications in high-risk patients with comorbidities, generating substantial costs to the city health system [3].

The aim of this study was to describe and compare the epidemiologic characteristics, management strategies and costs of varicella outbreaks in the HNRG from 2000 to 2019, before and after the introduction of the varicella vaccine.

## Material and methods

A retrospective, analytic study of the impact of nosocomial varicella outbreaks at the HNRG, based on nosocomial case notifications to the Active Epidemiologic Surveillance System from 2000 to 2019.

**Nosocomial varicella outbreaks** are defined as the presence of a clinical varicella case (patient, health care professional, or visitor) that occurs in hospital setting, which determines an inadvertent exposure and require the implementation of control measures.

**Significant exposure** to Varicella virus in hospital contacts is considered when sharing 2- to 4-bed rooms or adjacent beds in large rooms, face-to-face contact with a staff member or infectious patient, or visit by a person apparently contagious.

For exposed patients without evidence of immunity airborne and contact precautions from 8 until 21 days after exposure to the index patient or until 28 days after exposure for those who received VariZIG or intravenous immune IGIV.

During the study period the nosocomial varicella outbreak per year, their seasonality, demographic characteristics and comorbidities of index cases and contacts, laboratory diagnosis and medications for post-exposure prophylaxis to control spread of disease to susceptible individuals were analyzed. Medications for susceptible contacts comprised varicella vaccination, VarZIG), IGIV and antiviral drugs. Local and international post-exposure management guidelines were followed: [3–5]

1. Varicella vaccination for healthy immunocompetent individuals 1 year of age or older preferably within 3 to 5 days after exposure.
2. VarZIG for unvaccinated patients or individuals without a history of varicella within 10 days after contact with an index case, using recommended doses for immunocompromised patients, pregnant women and neonates. (VarZIG) must be administered intramuscularly, dosage: 62.5 units for infants weighing < 2 kg; 125 units 2 to10 kg; 250 units 10.1 to 20 kg, 375 units 20 to 30 kg; 500 units 30 to 40 kg and 625 units > 40 kg.
3. IGIV. Recommended dose 400mg/kg.
4. Acyclovir given within 7 to 10 days after exposure. 20 mg/kg per dose QID PO, maximum daily dose of 3200 mg for 7 days, evidence for this indication in children is limited.

We carried out a quasi-experimental impact analysis before (years 2000 to 2014) and after (2016 to 2019) varicella vaccine introduction to the NIP in 2015, transition year and excluded from the analysis taking into account the low vaccination coverage at the beginning of this strategy [6].

The number of outbreaks during prevaccination (PreV) and postvaccination (PostV) periods adjusted by all cause hospital discharges was analyzed as well as seasonality.

Analysis was performed comparing nosocomial varicella outbreaks rates (per 10,000 discharges) between both periods.

Epidemiological and clinical variables of index cases and contacts, and medication options for susceptible contacts were expressed as absolute values and percentages with 95% confidence intervals using OPEN EPI Program [7]. Imputation methods for missing values were not used, allowing for a certain degree of data inconsistency when disaggregating for individual categories.

Cost per susceptible case was estimated according to the prescription patterns of post-exposure prophylaxis to control spread of disease Table 3. Doses of different prophylaxis treatment were estimated for average 3.5 years old weight 15 KG (50^th^ percentile). Costs of treatments were expressed as absolute values in Argentine pesos AR$ and its equivalent in dollar (USD) according to an exchange 1USD = 81.25 AR$. The *outbreaks prophylaxis economic impact (OPEI)* after the incorporation of the vaccine was estimated using the following formula:

$$OPEI={\left(a\times b\times c\right)}_{preV}‐{\left(d\times e\times c\right)}_{postV}$$

a _=_ *average number outbreaks per year*; *b = average number of susceptible outbreaks per year; c = cost per susceptible case*; *PreV = Pre vaccination period*.

*Outbreaks prophylaxis economic impact*, *d = average number outbreaks per year; e = average number of susceptible outbreaks per year; c = cost per susceptible case PostV = Post* vaccination period.

We estimated the average cost for outbreak control based on medication patterns for management of susceptible contacts, using a model in Epi Info version 7 [8].

Ethics Statement: This is retrospective study approved by the Ricardo Gutierrez Research Ethic Committee, and all data was fully anonymized.

## Results

**Fig 1:**
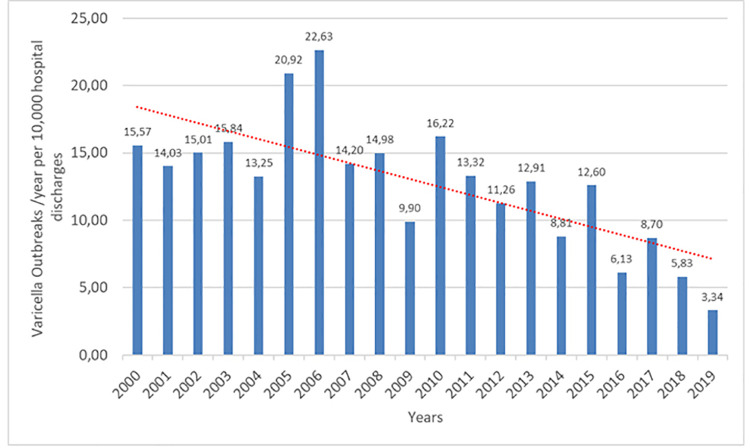
Varicella outbreaks/year person 10,000 hospitalization.

Between 2000 and 2019, 259 nosocomial outbreaks were reported affecting 2,711 individuals including index-cases and contacts; 237 outbreaks occurred during the PreV period with an average of 15.87 (13.91–18.02) per year, and 22 during the PostV period, average 5.5 per year (3.44–8.32) Fig 1.

**Fig 2:**
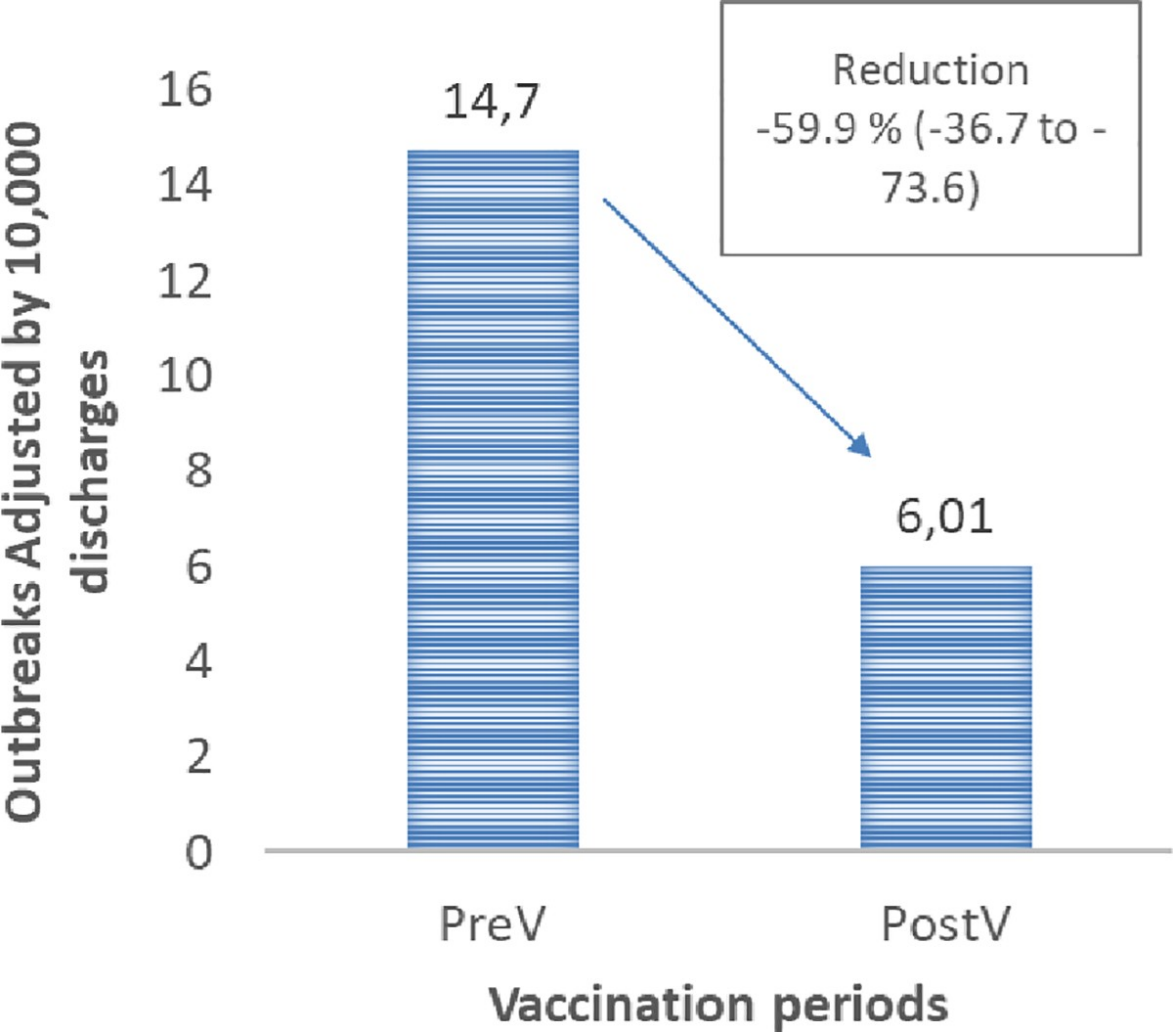
Outbreaks adjusted by 10,000 discharges.

When comparing PreV and PostV annual nosocomial outbreaks adjusted by all cause discharges we found a reduction of 59.13% (-36.68%, -73.62%) after vaccine introduction Fig 2.

**Fig 3:**
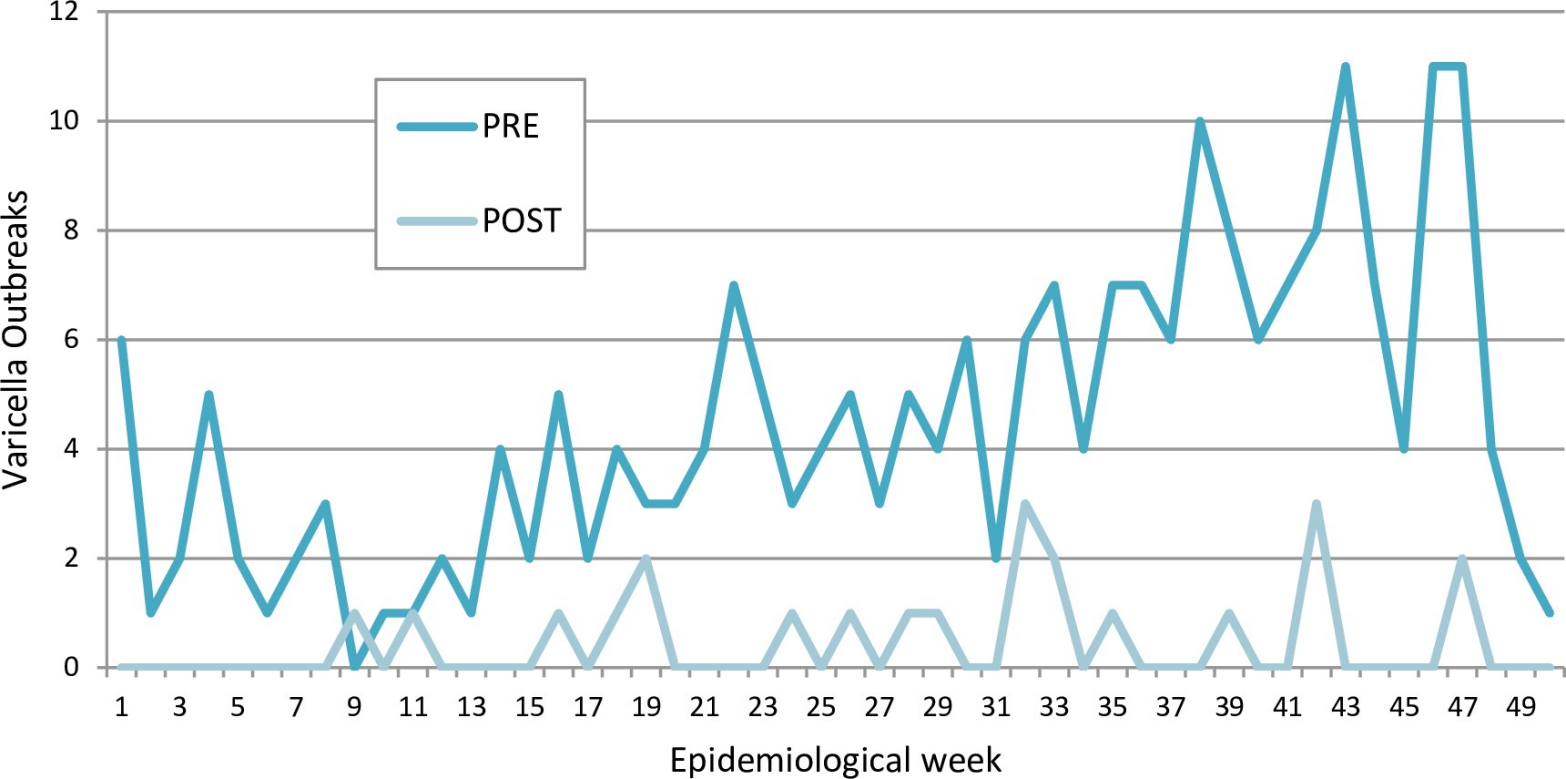
Seasonality patterns were observed during both periods.

Similar seasonality patterns were observed during both periods Fig 3.

Of a total of 259 outbreaks, 146 (56.45%) occurred in clinical wards, 55 (21.53%) in specialist units (surgery, day care facility and infectious diseases department), 54 (20.85%) in oncology and rheumatology unit waiting rooms, and 4 (1.5%) were categorized as miscellaneous.

Epidemiological and clinical characteristics of index cases and contacts are summarized in Tables 1 and 2.

**Table 1:** Epidemiological and clinical characteristics of index cases in pre- and post-vaccination periods.

Index cases *	PreV Period n = 237			Post V Period n = 22			p
	n	%	IC 95%	n	%	IC 95%	
Gender (male)	98/194	50.52	43.24–57.75	12/20	60	36.05–80,88	NS
Immunosupressed	46/117	39.32	30.41–48.77	9/22	40.91	20.71–63.65	NS
Comorbidities	51/96	53.04	45.69–60.98	11/21	52.38	28.78–74.29	NS

*Age (median, range) PreV Period was 36.5 months (17–72) and 34.5 months (14–72) Post Period.

**Table 2:** Epidemiological and clinical characteristics of contacts in pre- and post-vaccination periods.

Contacts *	Period (preV)			Period (postV)			p
	n = 2156	%	IC95%	n = 200	%	IC95%	
Gender (male)	898/1687	53.23	50.82–55.63	104/186	55.91	48.46 63.17	NS
Susceptible	1,184/1,785	66.33	64.08 68.51	172/199	86.43	80.88–90.86	<0,0001
Immunosupressed	680/1485	45.79	43.24–48.37	105/200	52.5	45.34–59.59	NS
Comorbidities	906/1105	81.99	79.57–84.19	131/198	66.16	59.11–72.72	<0,0001
Non susceptible	601/1785	33.66	31.5–35.89	27/199	13.56	9.32–18.87	< <0.0000001
Prior Vaccination	55/433	12.70	9.79–16.30	20/200	10.00	6.22–15.02	NS
History of varicella	411/1045	39.33	36.37–42.37	31/194	15.98	11.12–21.91	<0,0001
Positive serology	95/294	46.63	36.71–50.73	19/197	9.64	5.9–114.65	NS

*Age (median, range) PreV Period was 60 months (16–132) and 48 months (23–132) Post Period.

A total of 1,356 susceptible contacts were registered, 1,184 PreV and 172 PostV. The average number of susceptible contacts per outbreak was 5 (1,184/237) in the PreV and 7.81 (172/22) PostV. Serology testing for prior varicella infection was carried out in 166 contacts in the PreV period and 10 in the PostV period. Post-exposure medication use (vaccine, VZIG, acyclovir) for both periods is summarized in Table 3.

**Table 3:** Management of susceptible contacts pre- and post-vaccination.

Prescripton	Period PreV			Period PostV			p
	n	%	95%CI	n	%	95%CI	
Vaccination	184/658	27.96	24.63–31.49	30/130	23.07	16.44–30.90	NS
Immuno globulin	255/658	38.75	35.08–42.52	49/130	37.69	29.67–46.25	NS
Acyclovir	219/658	33.28	29.76–36.95	51/130	39.23	31.12–47.82	NS

No significant differences were observed related varicella prophylaxis medication between both periods.

The cost per prescription was estimated as: vaccination 500 AR$ (6.15 USD) Immune globulin 16,720 AR$ (205.78 USD) and Acyclovir 270 AR$.(3.33 USD) The cost per susceptible case (adjusted for the prescription rate described in the Table 3) during the PreV was estimated in 6,708 AR$ IC95% (6,068–7,366) (82.56: 74.68–90.6 USD) and PostV 6,522 AR$ IC 95% (5,866–8,016) (80.27:72.19–98.65 USD) Considering that during the preV period a total of 15.87 outbreaks per year were observed with an average of 5 susceptible cases per outbreak (15.87×6,708 $ ×5 = AR$532,280) (USD 6,551.13) and during the postV period 5.5 annual outbreaks were observed with an average of 7.8 susceptible per outbreak (5.5 ×AR$ $6,522×7.8 = AR$280,152), (USD 3,448) the economic impact on the reduction of intrahospital outbreaks after the incorporation of the vaccine, translated into an estimated average savings per year of AR$ -252,128 AR$ (USD-3103).

## Discussion

Nosocomial varicella outbreaks were dramatically reduced after varicella vaccine was introduced to the Argentine NIP However, the number of susceptible individuals exposed per outbreak in the PostV period increased.

Our results were consistent with other published studies in terms of outbreak reduction related varicella vaccination considering one or two doses.

Based on surveillance data published by Leung et al [9], the number of outbreaks declined by 78%, decreasing from 147 in 2005 to 33 outbreaks in 2012 after the routine 2-dose varicella vaccination program in US Civen et al^.^ [10] also showed that during a 10-year period (1995‒2005), in a population vaccinated with a one-dose schedule, outbreaks significantly decreased in number (from 236 to 46, p < .001), The results have been positive for both one and two doses of varicella vaccine, However, there is consensus that varicella vaccination decreases the number, size and duration of varicella outbreaks and that such decreases are even greater with a two-dose schedule [11].

Although outbreaks decreased, demographic characteristics of index cases, contacts and susceptible contacts were proportional similar for both periods.

As observed previously by Weber et al [12], most outbreaks in our study occurred when infected individuals visited the hospital during early phase of disease when it is highly contagious, or less often because of errors isolating patients with varicella retained in hospital waiting-rooms. We found health care workers were not susceptible to infection, probably because of exposure to varicella before vaccine introduction (over 95% of adults had experienced disease and developed immunity) [13]. There is also a prevention program at the HNRG providing vaccination against varicella to susceptible hospital workers. Between 2010 and 2018, 31 (14%) of a total of 221 healthcare workers showed no varicella antibodies on serology tests, similar to other reports that found susceptibility rates ranging between 1.9% to 10% among hospital workers after vaccine introduction [14,15].

Varicella zoster virus is highly contagious; the disease is particularly severe in immunocompromised patients which are why transmission in hospital setting causes such serious problems. Risk of transmission is also high in paediatric patients, seronegative adult patients and susceptible healthcare workers [16,17]. In this series we found that both in the PreV and PostV periods, around 50% of exposed individuals were immunocompromised patients, in agreement with the management options implemented to prevent the spread of disease. Varicella outbreaks also affect other patients, their family members and healthcare workers. Prevention measures must be implemented for unvaccinated individuals with no prior history of varicella or negative serologic tests [3,4].

For countries with varicella vaccination programs, WHO recommends vaccinating susceptible immunocompetent contacts to control outbreaks and prevent spread, prioritizing groups according to programme objectives and outbreak characteristics [18]. In the case of nosocomial outbreaks, vaccines must be provided to susceptible immunocompetent persons particularly those belonging to vulnerable populations with limited access to vaccines and health services in general [19].

Use of VZIG was the most expensive treatment generating significant impact on total costs both in the PreV and PostV periods. In this study we did not consider expenses attributed to hospitalization days of susceptible contacts, which would increase overall VZ related costs. In Argentina, Giglio et al estimated average cost of hospitalization due to varicella to be 2,464.6 USD per case (95% CI: 1949.7–2979.5) [20].

Our results show that susceptible contacts per outbreak increased in the PostV period. In spite of not having a clear explanation to the increased proportion of susceptible contacts in the PostV we may consider that the latest outbreaks occurred in critical areas where the majority of inpatients contacts were immunocompromised.

Although several authors have suggested susceptible contacts numbers may increase after vaccine introduction in a one-dose regimen [21], in our study the PostV was too short and most susceptible contacts (90%) in this period were not vaccinated^.^ Since immunization started in 2015 and given the fact that average age of contacts during the PostV period was 4 years, one must assume most contacts had not been vaccinated during this period and had not acquired natural immunity.

In this sense, Argentine Pediatrics Society recommends the introduction of a second dose to the National Immunization Program^.^ [22].

Our study presents limitations, information was lacking for some events and imputation of missing data was not performed to maintain validity of results.

Taking into account the different length of PreV and PostV, condition that could compromise the validity of our results, we adjusted nosocomial outbreaks by hospital discharges in order to reduce any potential bias On the other hand not all cost were added in this equation and overall economic impact of vaccine may be even higher.

Finally for susceptible subjects who received the vaccine, we didn`t include other program costs such as cold chain expenses and human resources related to the vaccine administration were supported by the government understanding that vaccination is a public health and the associated costs are more than just vaccine costs alone.

To our knowledge, no other studies on nosocomial varicella outbreaks in Latin America have been published to date, these reports would contribute to estimate the impact of varicella vaccination on an infrequently outcome described such us the prevention and control of outbreaks in hospital settings.

## Conclusions

The number of annual varicella hospital outbreaks at the HNRG decreased dramatically after varicella vaccine was introduced to NIP in Argentina. Seasonality remained unchanged and susceptible contacts per outbreak increased in the PostV period. The reduction in the number of annual outbreaks after the incorporation of varicella vaccination into to the Argentine National Immunization program has had a favorable economic impact.

## Supporting information

S1 File (DOCX)
