# Supplementary material for: Impact of varicella vaccine on nosocomial outbreaks and management of post exposure prophylaxis following in a paediatric hospital
Source: PLoS One. 2021 May 20;16(5):e0251496. doi: 10.1371/journal.pone.0251496 (PMC8136631; doi:10.1371/journal.pone.0251496)
Supplement: S1 File — (DOCX) [file pone.0251496.s001.docx]

| **Year** | **Outbreaks** | cumulative n | cumulative % | all cause hospital discharges | **Outbreaks** / all cause hospital discharges *10000 | |
| --- | --- | --- | --- | --- | --- | --- |
| 2000 | 16 | 16 | 6,2 | 10274 | 15,57 |  |
| 2001 | 15 | 31 | 12,0 | 10695 | 14,03 |  |
| 2002 | 17 | 48 | 18,5 | 11323 | 15,01 |  |
| 2003 | 18 | 66 | 25,5 | 11364 | 15,84 |  |
| 2004 | 15 | 81 | 31,3 | 11322 | 13,25 |  |
| 2005 | 23 | 104 | 40,2 | 10996 | 20,92 |  |
| 2006 | 23 | 127 | 49,0 | 10162 | 22,63 |  |
| 2007 | 14 | 141 | 54,4 | 9856 | 14,20 |  |
| 2008 | 14 | 155 | 59,8 | 9344 | 14,98 |  |
| 2009 | 9 | 164 | 63,3 | 9087 | 9,90 |  |
| 2010 | 17 | 181 | 69,9 | 10482 | 16,22 |  |
| 2011 | 13 | 194 | 74,9 | 9758 | 13,32 |  |
| 2012 | 11 | 205 | 79,2 | 9771 | 11,26 |  |
| 2013 | 12 | 217 | 83,8 | 9298 | 12,91 |  |
| 2014 | 8 | 225 | 86,9 | 9082 | 8,81 |  |
| 2015 | 12 | 237 | 91,5 | 9522 | 12,60 |  |
| 2016 | 6 | 243 | 93,8 | 9795 | 6,13 |  |
| 2017 | 8 | 251 | 96,9 | 9197 | 8,70 |  |
| 2018 | 5 | 256 | 98,8 | 8576 | 5,83 |  |
| 2019 | 3 | 259 | 100,0 | 8990 | 3,34 |  |
| **Total general** | **259** |  |  |  |  |  |

| Tipo de Case: =Contact | |  |  |  |  |
| --- | --- | --- | --- | --- | --- |
| Year | Frequency | Percent | Cum. Percent | Fleiss 95% LCL | Fleiss 95% LCL |
| 2000 | 252 | 10,28% | 10,28% | 9,12% | 11,56% |
| 2001 | 183 | 7,46% | 17,74% | 6,47% | 8,59% |
| 2002 | 292 | 11,91% | 29,65% | 10,67% | 13,27% |
| 2003 | 237 | 9,67% | 39,31% | 8,54% | 10,92% |
| 2004 | 77 | 3,14% | 42,46% | 2,50% | 3,93% |
| 2005 | 168 | 6,85% | 49,31% | 5,90% | 7,94% |
| 2006 | 126 | 5,14% | 54,45% | 4,31% | 6,11% |
| 2007 | 91 | 3,71% | 58,16% | 3,01% | 4,56% |
| 2008 | 113 | 4,61% | 62,77% | 3,83% | 5,53% |
| 2009 | 89 | 3,63% | 66,39% | 2,94% | 4,47% |
| 2010 | 176 | 7,18% | 73,57% | 6,20% | 8,29% |
| 2011 | 106 | 4,32% | 77,90% | 3,57% | 5,22% |
| 2012 | 121 | 4,93% | 82,83% | 4,13% | 5,89% |
| 2013 | 85 | 3,47% | 86,30% | 2,79% | 4,29% |
| 2014 | 40 | 1,63% | 87,93% | 1,18% | 2,24% |
| 2015 | 96 | 3,92% | 91,84% | 3,20% | 4,78% |
| 2016 | 58 | 2,37% | 94,21% | 1,82% | 3,07% |
| 2017 | 57 | 2,32% | 96,53% | 1,78% | 3,02% |
| 2018 | 59 | 2,41% | 98,94% | 1,85% | 3,11% |
| 2019 | 26 | 1,06% | 100,00% | 0,71% | 1,57% |
| TOTAL | 2452 | 100,00% | 100,00% |  |  |

|  | susceptible | | |
| --- | --- | --- | --- |
|  | TOTAL | n Outbreaks | rate |
| PRE | 1184 | 225 | 5,26 |
| POST | 172 | 22 | 7,81818182 |

| prescription | Frequency | Percent | Cum. Percent | Fleiss 95% LCL | Fleiss 95% LCL |
| --- | --- | --- | --- | --- | --- |
| 0 | 56 | 4,15% | 4,15% | 3,18% | 5,39% |
| 1 | 126 | 9,34% | 13,49% | 7,87% | 11,05% |
| 2 | 227 | 16,83% | 30,32% | 14,89% | 18,96% |
| 3 | 324 | 24,02% | 54,34% | 21,78% | 26,41% |
| 4 | 289 | 21,42% | 75,76% | 19,28% | 23,73% |
| 5 | 173 | 12,82% | 88,58% | 11,11% | 14,75% |
| 6 | 154 | 11,42% | 100,00% | 9,79% | 13,26% |
| TOTAL | 1349 | 100,00% | 100,00% |  |  |
|  | 95,2683616 |  |  |  |  |

| Año | outbreaks/year/ 10,000 all cause hospital discharges | | |
| --- | --- | --- | --- |
| 2000 | 15,57 |  |  |
| 2001 | 14,03 |  |  |
| 2002 | 15,01 |  |  |
| 2003 | 15,84 |  |  |
| 2004 | 13,25 |  |  |
| 2005 | 20,92 |  |  |
| 2006 | 22,63 |  |  |
| 2007 | 14,20 |  |  |
| 2008 | 14,98 |  |  |
| 2009 | 9,90 |  |  |
| 2010 | 16,22 |  |  |
| 2011 | 13,32 |  |  |
| 2012 | 11,26 |  |  |
| 2013 | 12,91 |  |  |
| 2014 | 8,81 |  |  |
| 2015 | 12,60 |  |  |
| 2016 | 6,13 |  |  |
| 2017 | 8,70 |  |  |
| 2018 | 5,83 |  |  |
| 2019 | 3,34 |  |  |

|  |  |  | |  | |  |
| --- | --- | --- | --- | --- | --- | --- |
| \|  \| \| --- \| |  |  | |  | |  |
|  |  |  | |  | |  |
|  |  |  | |  | |  |
|  |  |  | |  | |  |
|  |  |  | |  | |  |
|  |  |  | |  | |  |
|  |  |  | |  | |  |
|  |  |  | |  | |  |
|  |  |  | |  | |  |
|  |  |  | |  | |  |
|  |  |  | |  | |  |
|  |  |  | |  | |  |
|  |  |  | |  | |  |
|  |  |  | |  | |  |
|  |  |  | |  | |  |
|  |  |  | |  | |  |
|  |  |  | |  | |  |
|  |  |  | |  | |  |
|  |  |  | |  | |  |
|  | | | |  | |  |
|  | | | |  | |  |
|  | | | |  | |  |
|  | | | |  | |  |
|  | | | |  | |  |
|  | | | |  | |  |
|  | | | |  | |  |
|  | | | |  | |  |
|  | | | |  | |  |
